# The study protocol of the Norwegian randomized controlled trial of electroconvulsive therapy in treatment resistant depression in bipolar disorder

**DOI:** 10.1186/1471-244X-10-16

**Published:** 2010-02-23

**Authors:** Ute Kessler, Arne E Vaaler, Helle Schøyen, Ketil J Oedegaard, Per Bergsholm, Ole A Andreassen, Ulrik F Malt, Gunnar Morken

**Affiliations:** 1 Moodnet Research Group, Haukeland University Hospital, Bergen, Norway; 2 Department of Clinical Medicine, Section of Psychiatry, University of Bergen, Norway; 3 Moodnet Research Group, Stavanger University Hospital, Stavanger, Norway; 4 Psychiatric Clinic Førde, Førde, Norway; 5 Division of Psychiatry, Oslo University Hospital, Ullevål, Oslo, Norway; 6 Institute of Psychiatry, University of Oslo, Oslo, Norway; 7 Oslo University Hospital, Rikshospitalet, Oslo, Norway; 8 Østmarka Psychiatric Department, St Olav University Hospital, Trondheim, Norway; 9 Department of Neuroscience, Norwegian University of Science and Technology, Trondheim, Norway

## Abstract

**Background:**

The treatment of depressive phases of bipolar disorder is challenging. The effects of the commonly used antidepressants in bipolar depression are questionable. Electroconvulsive therapy is generally considered to be the most effective treatment even if there are no randomized controlled trials of electroconvulsive therapy in bipolar depression. The safety of electroconvulsive therapy is well documented, but there are some controversies as to the cognitive side effects. The aim of this study is to compare the effects and side effects of electroconvulsive therapy to pharmacological treatment in treatment resistant bipolar depression. Cognitive changes and quality of life during the treatment will be assessed.

**Methods/Design:**

A prospective, randomised controlled, multi-centre six- week acute treatment trial with seven clinical assessments. Follow up visit at 26 weeks or until remission (max 52 weeks). A neuropsychological test battery designed to be sensitive to changes in cognitive function will be used. Setting: Nine study centres across Norway, all acute psychiatric departments. Sample: n = 132 patients, aged 18 and over, who fulfil criteria for treatment resistant depression in bipolar disorder, Montgomery Åsberg Depression Rating Scale Score of at least 25 at baseline. Intervention: Intervention group: 3 sessions per week for up to 6 weeks, total up to 18 sessions. Control group: algorithm-based pharmacological treatment as usual.

**Discussion:**

This study is the first randomized controlled trial that aims to investigate whether electroconvulsive therapy is better than pharmacological treatment as usual in treatment resistant bipolar depression. Possible long lasting cognitive side effects will be evaluated. The study is investigator initiated, without support from industry.

**Trial registration:**

NCT00664976

## Background

Bipolar disorder (BD) is a psychiatric disorder with a prevalence of 1.7 to 3.7 percent in the adult population [1], characterized by periods of severe affective symptoms with normal periods in between. BD often has an unfavourable outcome [2]. There are two subtypes, Bipolar I and Bipolar II, and depression is arguably a more important facet of both types [3,4]. Depressive episodes are more numerous, last longer, and most suicides occur during these periods [5].

In contrast to the manic phases the treatment options for the depressive phases are poor. Antidepressants have small if any effect in bipolar depression [6], lithium is not very effective [7], there are a few antipsychotics with effect [8,9], but there are some indications for effect of mood stabilizers [10]. The mainstay of current specialist treatment in Norway is a combination of a mood stabilizer, antipsychotic and/or an SSRI or an SNRI. In the last few years the use of the anticonvulsant lamotrigine has become common. Such a combination is in agreement with the recommendations in the treatment guidelines [11]. Electroconvulsive therapy (ECT) was for decades a controversial treatment, but the patients' acceptance of this treatment has increased, leading to an increase in its use [12]. However, ECT resources are limited, and lack of availability often means that it is not a real treatment option. In Norway, the ECT service is fairly good at all regional hospitals. For the most severely ill and treatment resistant BD patients, ECT is considered to be the most effective treatment [11,13]. However, this recommendation is based on clinical experience, as no RCT of ECT in this disorder has been performed. Thus, neither effect size nor comparative efficacy is known [11,14]. This basic lack of knowledge was the motivation for initiating the present study.

No systematic attempts have been made to define what constitutes refractoriness in bipolar depression, and several definitions are used in the literature. In unipolar depression, treatment resistance is present when two or more antidepressive psychopharmacological treatment options have been adequately tried [15]. In the present study with patients suffering from BD, we have used a pragmatic approach and defined refractory bipolar depression as depression that failed to respond to two trials (during lifetime) with antidepressiva and/or mood stabilizer with proven efficacy in bipolar depression (lithium, lamotrigine, quetiapine, olanzapine) in adequate doses for at least 6 weeks or until cessation of treatment due to side effects.

There is little empirical evidence for drug treatment of treatment resistant bipolar depression. Reflecting this, the American Psychiatric Association Treatment Guidelines for BD have a low confidence for their recommendations in this area [11]. The combination of an atypical antipsychotic with documented antidepressant effect and an antidepressant receives the highest grade of recommendation as does ECT.

Patients with BD have specific cognitive impairments affecting a variety of cognitive domains, not only in depressive phases [16-18]. There is little knowledge how cognitive dysfunction changes with treatment of depression. We will use well validated instruments to assess the most important and clinically relevant cognitive domains at baseline and changes during the treatments.

ECT is associated with cognitive side effects [13], but there are few studies comparing cognitive impairment in drug- and ECT-treated patients. The severity, type and duration of the cognitive dysfunction seems to dependent on methods used in ECT administration [19-21]. Given optimal methodology recent studies have found improved global cognitive function as assessed by Modified Mini-Mental State Examination (mMMS) [22] and verbal learning 6 months after completion of an ECT treatment compared to baseline [21]. In the same study, autobiographical memory was impaired at six months after the completion of ECT treatment if ECT was administered by bitemporal electrode placement. Further studies are needed to evaluate possible lasting cognitive side effects of ECT. No studies have evaluated cognitive impairment in patients with bipolar depression treated with pharmacological treatments compared to ECT in a randomised setting.

Health-related quality of life measures have become increasingly important as a type of patient-reported outcome documenting the subjective psychosocial burden associated with chronic illness.

In patients with major depression ECT is widely acknowledged as an effective and appropriate acute treatment. Still questions remain regarding whether ECT is associated with a net improvement in function and quality of life. In recent guidelines from United Kingdom [23] ECT use was restricted until more information becomes available about its effects on memory and quality of life.

A growing body of evidence suggests that inflammation may be linked to depression [24-26]. There are indications that cytokines may cause depressive episodes [27]. Few studies have investigated the immune system in BD. One study found that BD is associated with increased production of the pro-inflammatory cytokines, both in the manic and depressed phase (IL-8 and TNF-α) compared to healthy subjects [28].

Two recent reviews [29,30] concluded that inflammation appears relevant to BD across several important domains and proposed that TNF-alpha modulation is a target for disease-modifying treatment of BD. Further research is warranted to investigate the reciprocal associations between inflammation and symptoms, comorbidities, and treatments in BD.

The aim of the current study is to document the effect size, relative effect size and adverse effects of ECT compared to treatment as usual in treatment resistant bipolar depression. We want to assess the cognitive function in bipolar depression, and how ECT and treatment as usual affect cognitive functioning. Furthermore possible inflammation processes involved in bipolar depression will be investigated. The design enables us to assess changes in a wider spectrum of cytokines as a function of treatment modality and changes in clinical status.

## Methods

### Study design

**Figure 1:**
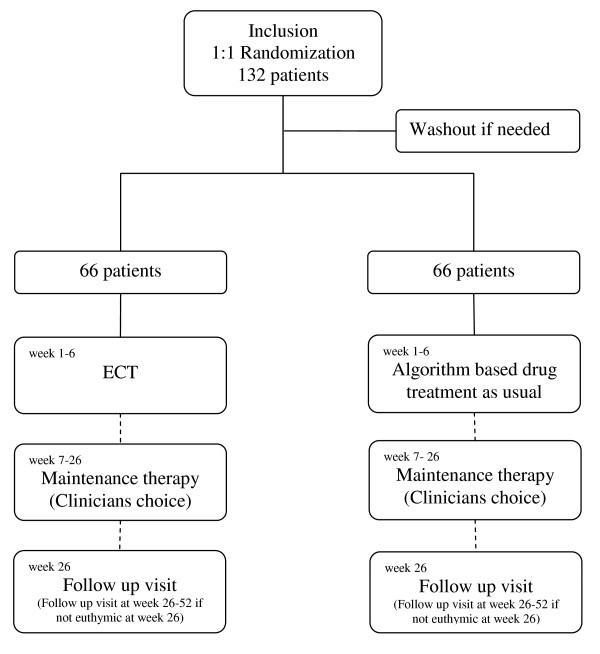
**Flow Chart of Study Design**.

This trial is a randomised controlled multi-centre study aimed to study efficacy and cognitive side effects of ECT for the treatment of treatment resistant depression in BD, compared to combined pharmacological treatment, including the MAOIs tranylcypromine and phenelzine. A flow chart of the study design is shown in Fig. 1.

Relevant patients with depression are addressed in order to establish whether they are willing to be screened for the study. The patients must be assigned a patient number and sign the consent form after receiving oral and written information about the study prior to undergoing any study procedures.

Each treatment trial lasts 6 weeks. The medication that would be used if a patient should be randomized to drug treatment will be determined at enrolment, before randomisation. If a patient receives ECT and reaches remission earlier than six weeks, the ECT treatment is terminated and the patient is switched to maintenance therapy. If a patient or the treating clinician decides that the patient could receive better treatment outside of the study, the patient may leave the study at all times, as specified in the informed consent. In case of remission after the six weeks trial the patient continues with maintenance drug treatment at the clinicians decision, guided by relevant algorithm as for example in Goodwin & Jamison 2007 [31]. In case of non- remission the clinician might consider the other treatment condition. This will not be part of the present study.

### Subjects

#### Trial sample and recruiting centres

132 patients fulfilling the criteria below will be included into this study by nine centres in Norway.

All the patients will first be included in the "Bipolar Research And Innovation Network, Norway" (BRAIN) study, a multicenter study describing bipolar disorder patients in Norway. This study is approved by The Regional Committee for Medical and Health Research Ethics, Middle - Norway. The patients will give written informed consent both to the BRAIN study and the ECT-study.

#### Inclusion and exclusion criteria

The inclusion criteria were designed to ensure that the included patients are typical for patients referred to ECT in Norway in order to have results that are clinically significant, while the exclusion criteria are designed to ensure the patients' safety. Inclusion and exclusion criteria are shown in table 1 and 2.

**Table 1:** Inclusion criteria

Diagnosis of DSM-IV-TR [52] of Bipolar I or Bipolar II disorder as verified by the semi-structured diagnostic interviews SCID [37] or MINI plus [36]. The diagnosis may be supported by information from significant others, and from hospital records. Angst's hypomania checklist [53] is used to increase the detection of hypomanic symptoms. SCID or MINI plus will be used to diagnose the patient.
ECT is indicated.
Severity: meet DSM-IV-TR criteria of depressive episode, MADRS [40] of 25 or above
Treatment resistance: None response to two trials (during lifetime) with mood stabilizers with proven efficacy in bipolar depression (lithium, lamotrigine, quetiapine, olanzapine) and/or antidepressants.
A trial is defined as at least 6 weeks in adequate or tolerated dose as reported by the patient, or patients that have been unable to comply with 6 weeks trials of mood stabilizer or an antidepressant.
None response: Less than 50% reduction in MADRS values or still meet DSM -IV-TR criteria of depressive episode
Inpatients the first week after start of treatment condition
The patients are to be treated by the psychiatrist at the hospital for the whole duration of the study (6 weeks)
Age ≥ 18
Patient competent to give informed consent according to the judgement of the clinician
Written informed consent
Patient sufficiently fluent in Norwegian language to ensure valid responses to psychometric testing (for patients enrolled to neuropsychological assessment: Norwegian as primary language or 12 years attendance of a Norwegian school)

**Table 2:** Exclusion Criteria

Earlier ECT none response
ECT within the last six months
Rapid cycling BD (e.g.4 or more episodes per year)
Use of medication or substances (such as pethidine, alcohol, drugs) incompatible with treatment (medication or ECT). Such medication must be stopped a least 5 half-lives before start of treatment.
Current use of all other psychotropic medication during the study period with the exception of the following: The use of alimemazine (max dose 30 mg daily), chlorpromazine (max dose 25 mg × 2 daily) and chlorprothixene (max dose 20 mg × 2) is allowed. The use of mianserine (max dose 10 mg daily) is allowed. Medication related to the ECT procedure is allowed.
For Medication in control group refer to Medication in control group - Treatment As Usual
Inability to comply with study protocol
Unstable serious medical conditions, including clinically relevant laboratory abnormalities
Conditions that affect neuropsychological assessment such as Parkinson's Disease, Multiple sclerosis, stroke, alcohol and substance abuse or dependence (according to SCID or DSM-IV-TR)
Fertile women without adequate contraception (Adequate contraception includes: abstinence, oral contraceptives, intrauterine devices, barrier method)
Young Mania Rating Scale (YMRS) [43] of 20 or more
Patient at high suicidal risk according to clinicians' judgement

#### Withdrawal criteria

The patient will be withdrawn from the study if the clinician finds that the patient is in need of, or better served with other treatment, or if Exclusion criteria are met. The patient will also be withdrawn from the study if the clinical condition gets significantly worse according to the clinicians' judgement or if patients withdraw their consent. The date and the reason for discontinuation are noted. All patients prematurely discontinuing the trial must be seen for a final evaluation.

### Inclusion, randomization and masking of study groups

All patients suffering from a major depressive episode admitted to one of the nine study centres are evaluated for inclusion. During the screening the local participating psychiatrist will determine whether the patient fulfils the inclusion criteria and none of the exclusion criteria. Patients will be randomly assigned to the two groups. The randomization is stratified separately in each study centre. The patient and treating psychiatrist are unmasked about treatment modality. The assessments of depressive symptoms before and after ECT will be audio-taped. The audio-tapes will be co-rated by trained study personnel who are not involved in the treatment of the study patients. The analyses will be preformed on audio taped scores. There might be some patients who do not accept audio taping, in these cases investigator-rating will be used. Neuropsychological assessment is performed by trained test assistants who are blinded about treatment modality.

### Description of treatment

#### Method of assigning patients to treatment groups/Randomization

Patients' eligibility will be established before randomization to one of the treatment options. Relevant patients with BD are addressed in order to establish whether they are willing to be screened for the study. The patients must be assigned a patient number and sign the consent form after receiving oral and written information about the study prior to undergoing any study procedures. Patients will be randomized strictly sequentially, as patients are eligible for randomization. If a patient discontinues from the study, the patient number will not be reused, and the patient will not be allowed to re-enter the study.

We perform a stratified randomization separately for each centre using the default random number generator of SPSS 15 with a random seed. The randomization lists are kept concealed from the investigators.

After randomization patients will enter a wash out phase if necessary. The recommended time for patients to be without concomitant medication in contradiction to the study protocol is 5 t 1/2 for patients randomized to ECT and a varying time for patients randomized to treatment as usual.

#### ECT

##### Equipment

ECT will be administered with a Thymatron System IV Somatics Inc. or MECTA 5000 or 4000 models, providing brief-pulse, square wave, constant current.

##### Anaesthesia

Preferably the short acting anaesthetic thiopental will be used to obtain anaesthesia. Anaesthetic dose should be kept to a minimum (1.5-2.5 mg/kg iv). A dose large enough to inhibit ciliary reflex is usually larger than needed for ECT. Excessive anaesthetic dosage may raise seizure threshold and shorten seizure duration. The appropriateness of dosage will be determined at each treatment and adjustments made at subsequent treatments. Other anaesthetics may be used if indicated. Succinylcholine in a dose of 0.5 - 1.0 mg/kg iv will be used as a muscle relaxant. All patients will be hyperoxygenated during treatment. Patients breathe oxygen enriched air 1 to 2 minutes before and during the initiation of anaesthesia. Pulse oximetry will be used. Hyperventilation should be maintained even with high oxygen saturation. Use of other medication necessary during anaesthesia (e.g. for premedication or termination of prolonged seizure) is at the decision by the anaesthesiologist.

##### Stimulation electrodes placement

Stimulation electrodes will be placed ad modem d'Elia [32] (Right unilateral electrode placement, RUL). It is well documented that high dosage ECT with unilateral placement of stimulation electrodes is as effective as bilateral placement [19,20]. If the stimulation electrodes are placed on the non-dominant side, unilateral stimulation is associated with less cognitive impairment than bilateral stimulation [19,21]. In this study electrodes will be placed over the right hemisphere.

##### Stimulus

Using ultra-brief stimulation pulses (0.5 ms or less) is more effective and associated with less cognitive impairment than longer pulses and sinus current. The duration of the stimulus pulse in our study will be 0.5 ms. In the present study the initial stimulus energy will be determined by an aged based method, where the energy (E) is calculated as following [33]: Patient's age in years × 5 ≅ stimulus charge in mC. The Thymatron delivers a charge of 25.2 to 504 mC in 20 equal steps, set by the % Energy dial. According to the above formula this makes: Patient's age in years ≅% Energy. In order to consider gender specific differences in seizure threshold the % Energy will be adapted as following: For male patients: % Energy + 5 to 10%. For female patients: % Energy - 5 to10%. If there is not obtained a sufficient seizure in one session determined by clinicians decision (based on seizure duration, δ-waves and clinical effect) the patient may be restimulated in the same session or/and stimulus parameter will be adjusted in next session. The treatment should be followed by a comatose state, from which consciousness is gradually regained [34].

##### Medication after seizure

In case of postictal delirium either midazolam (0.5 - 2.5 mg iv) or thiopental (half of anaesthetic dose) may be administered. Postictal headache may be treated with paracetamol 1000 mg or ibuprofen 400 mg (can be repeated). Metoclopramide 10 mg tablets or iv in case of nausea.

##### Duration of treatment

Three sessions per week for up to six weeks, a total of up to 18 sessions. The ECT series may be tapered off when response is achieved by gradually increasing the intervals between treatments.

##### Quality Control

The ECT instruments will be regularly tested for effects and calibrated.

##### Registration

Each ECT session will be registered with the parameters listed in table 3.

**Table 3:** Parameters recorded after each ECT-session

Anaesthetic drugs used
Energy (%)
Charge delivered
Motor activity
Seizure duration
Postictal suppression index
Sustained coherence
Sustained power
Seizure energy index
Quality of δ-waves, seizure ending and postictal supression
Basal and peak heart rate
Time to reorientation (personal data, time, place)
Mood state immediate after recovery of orientation and the following hours, expressed like "good, neutral, don't know, better, changed" or "bad, depressed, no better, no change, feeling of not having had a treatment

##### Maintenance-treatment after ECT-course

At the end of course of ECT each patient will be given maintenance medication after clinicians' decision following treatment guidelines described elsewhere [31].

#### Medication in control group - Treatment as Usual

All participating hospitals use the treatment algorithm according to Goodwin and Jamison [35] adapted to Norwegian conditions as their routine treatment for bipolar depression. See Additional file 1. In addition the use of alimemazine (max dose 30 mg daily), chlorpromazine (max dose 25 mg × 2 daily), chlorprothixene (max dose 20 mg × 2), mianserine (max dose 10 mg daily) is allowed. In the control group the use of alopam 15 mg up to 3 times/day, zolpidem 10mg or zoplicone 7,5 mg is allowed as part of treatment as usual.

##### Wash out Phase

The recommended time for patients to be without concomitant medication in contradiction to the study protocol is 5 t 1/2 for patients randomized to ECT and a varying time for patients randomized to treatment as usual.

##### Randomized treatment Phase - treatment regimens

For treatment regimes see Additional file 1.

The treatment algorithm according to Goodwin and Jamison [35] is to be followed step by step. It is however possible for the clinician to depart from the stepwise use of the algorithm if they out of their knowledge to the patient consider one of the medicaments unacceptable or of no use to the patient. Patients that experience intolerable side effects on one medication listed in the algorithm may be switched to the next treatment option according to the algorithm.

##### Continuation phase

After the 6 weeks randomized treatment phase, the patients continue with medication recommended by their clinician guided by a relevant treatment algorithm as for example [31].

##### Treatment compliance

Compliance will be assessed based on patient's record of compliance (at each visit 3-10) as well as by serum level monitoring at week 3.

##### Concomitant Therapy

All medications (prescriptions or over the counter medications) continued at the start of the trial, or started during the trial and different from the trial medication must be documented. Patients, who receive ECT during the follow- up period, will be excluded from further analyses of neuropsychological functioning.

### Sample size calculation

#### A-priori power calculations

The primary response variable, change in MADRS-score between baseline and endpoint, will be done with the two-sample t-test at the significance level α = 0.05. The least clinical important difference between the treatment group (ECT) and the reference group (drug) to be detected was judged to be 4 in favour of ECT, and a SD = 7 for the change in MADRS is assumed. The possibility of an opposite effect could not be ignored and a two-sided test will be done. Thus the competing hypotheses are:

H0: Mean change in MADRS is the same in both groups vs.

HA: Mean change in MADRS is different in the two groups.

To detect a difference of 4 in mean change in MADRS between the two groups with a power of Π = 0.90 a total of n = 132 patients will have to be included in the analysis. Standard deviations of S= 6-7 are typical for MADRS in studies of patients with BD. The standard deviation for change in score is S√[2(1-R)] where R is the correlation between the two timepoints. Thus, with a correlation of at least 0.5 the standard deviation for the change will be at most S and the power at least 0.90.

Concerning changes in inflammation measures, effects sizes of more than 20-50% are expected, but standard deviations in this population will vary for different cytokines, and a formal power analysis has not been performed.

#### Statistical analysis

Response and remission rates will be compared using Chi Square tests. Secondary outcomes and cognitive function will be analysed with multiple regression analyses and other suitable statistical methods. ANOVAs and t-test will be made on a LOCF basis. Categorical variables will be analysed using chi square tests. Ordinal variables will be analysed using non-parametric tests in addition to using T tests and ANOVA. Time to remission and/or response will be analysed using survival analysis. The statistical analysis will be done by the scientific research team with assistance from experts in statistics. All patients who receive at least one medication with study medicine or ECT will be included in the analysis of the safety, demographic and baseline characteristic data. An analysis of treatment-emergent adverse events will be performed. All subjects who receive at least one medication with study medicine and provide post - baseline efficacy measurements will be included in efficacy data analyses (intention to treat). We will use last observations carried forward for subjects who leave the study prematurely. Analyses only including patients fulfilling the treatment will also be done. Baseline for all analyses is visit 2 (start of drug or ECT treatment).

## Assessments

An overview of variables is shown in table 4.

**Table 4:** Variable overview

	Study visit	1	2	3	4	5	6	7	8	9	10	Comment
	Week	-5t_1/2_	0	1	2	3	4	5	6	26	**30-52 **^1)^	^1) ^in euthymic phase
	Test											
	Informed consent	X										
Diagnostic interview	SCID or MINI plus	X										
	Inclusion/exclusion criteria	X										
NORBRAIN Entry Questionnaire NEQ	Socio demography Medical history etc	X										
	PANSS	X										
	GAF	X							X	X	X	
	Clinical examination	X										
	Current and concomitant medication	X	X	X	X	X	X	X	X	X	X	
Health related Quality of Life	SF 36	X							X	X	X	
Efficacy	MADRS	X	X^2)^	X	X	X	X	X	X^2)^	X	X	^2) ^audio taped
	IDS	X	X^2)^	X	X	X	X	X	X^2)^	X	X	^2) ^audio taped
	CGI-BP	X	X	X	X	X	X	X	X	X	X	
	PGI-I			X	X	X	X	X	X	X	X	
	YMRS	X	X	X	X	X	X	X	X	X	X	
	MSIF	X								X	X	
Relapse	Interview									X	X	
Overall cognitive function	MMS ^3)^		X	X	X	X	X	X	X			^3) ^in ECT-group only
Neuropsychological assessment		X							X	X	X	
	EMQ	X							X	X	X	
Adverse events	Interview UKU		X^4)^			X			X			^4) ^previous med.
	SAE-form			X	X	X	X	X	X			
Lab	Cytokines	X							X	X	X	
	Cortisol ^5)^								X			^5) ^morning cortisol in blood
	Other, incl cortisol^6)^	X										^6) ^as specified in Blood samples
	ECG ^7)^	X										^7) ^if indicated
	EEG	X										
	MRI caput	X										
Substance abuse	Urin	X										
Pregnancy	Urin ^8)^	X										^8) ^fertile woman
Compliance	Interview			X	X	X	X	X	X	X	X	
Compliance	Blood sample					X						
Temperament	TEMPS-A									X		
Migrene	Interview	X								X		

### Initial Subject and Disease Characteristics

Diagnosis is made on the basis of clinical interview and verified by the MINI-International Neuropsychiatric Interview (MINI) [36] plus or The Structured Clinical Interview for DSM-IV Axis I Disorders (SCID-I) [37]. All the patients are described by a modified version of Stanley Foundation Bipolar Network Entry Questionnaire (NEQ) at baseline [38,39].The first part of the questionnaire elicits data regarding vocational, educational, and economic status, onset and course of illness, family history, and past treatment. The second part assesses cycling and seasonal patterns, medical problems, medications (past and present), ability to function and symptomatic status, precipitants of illness (e.g. substance use), treatment adherence, and insight into the illness [38]. History of ECT is also assessed. At baseline symptom intensity will be measured by MADRS [40], Inventory of Depressive Symptoms (IDS) [41], Clinical Global Impression bipolar (CGI-BP) [42], and YMRS [43]. Global Functioning will be assessed with Global assessment of functioning (GAF) [44]. Health-related quality of life will be assessed with SF-36 [45]. There will be performed a medical examination, MRI Caput, ECG (if indicated), EEG, and laboratory tests. Substance abuse will be assessed by interview and urine test. Fertile woman are tested for pregnancy.

### Efficacy

#### Primary response variable

Patients will be interviewed at start (visit 2), weekly on week 1-5 (visit 3-7), on week 6/endpoint (visit 8), on a follow-up-visit after 26 weeks (visit 9) and in case of depressive, manic or mixed symptoms at weeks 26 on a control-visit between 26-52 weeks (visit 10). Visit 10 should be performed in euthymic state if possible. Patients will be interviewed using MADRS. The primary response variable is change in total MADRS-score at endpoint versus baseline.

#### Secondary response variables

IDS [41], CGI-BP [42]), GAF [44], Patient Global Impression of Improvement (PGI-I),

YMRS [43], SF-36 [45], Multidimensional Scale of Independent Functioning- MSIF [46], proportion in each group attaining remission, time to remission, proportion in each group attaining response, time to response, relapse (new episode).

##### Definitions

Response: at least a 50% reduction of the baseline MADRS score.

Remission: MADRS score of 12 or less at the end of trial.

New episode: A patient who has previously responded to treatment meets the DSM-IV criteria for Major Depressive Episode or Manic episode.

### Assessment of trait-like characteristics

The Temperament Evaluation of Memphis, Pisa, Paris and San Diego-auto questionnaire (TEMPS-A) [47] will be assessed at follow up and migraine diagnostic [48] pre-treatment and at follow up (week 26).

### Safety assessment

Safety data will include:

Clinical examination; electrocardiogram (if indicated); blood tests as described below (supplemented by others if clinically indicated) at start; urine test for substance abuse: amphetamine, cannabis, opiates, benzodiazepines, cocaine, methadone, pcp, zopiclone, buprenorphine, carisoprolol.

MMS weekly first six weeks in ECT-group to assess global cognitive functioning.

### Blood samples

A routine battery of blood measures will be analyzed at the local hospital biochemistry units:

Leucocytes, haemoglobin, ESR, Na +, K+, creatinine, glucose and triglyceride (blood samples were drawn after an overnight fast of at least 8 hours), cholesterol, methylmaleonic acid, albumin, Aspartate aminotransferase (AST), Alanine aminotransferase (ALT), Alkaline phosphatase (ALP), serum calcium, phosphate, vitamin B12, serum iron, serum ferritin, c-reactive protein, thyroid-stimulating hormone, free serum thyroxine, CDT, cortisol, homocysteine, folic acid, anti TPO, cyp 2D6 and cyp 2C9.

Serum samples for inflammation markers are shipped to long-term storage in - 80°C freezer. After all baseline samples are collected, the first series of analyses will be performed with appropriate methods, such as enzyme immunoassays (EIAs). Then the rest will be analyzed after the different time points have been reached.

The patients are included in the BRAIN study and tests for genetic analyses are done according to the BRAIN protocol earlier approved by the Regional Ethics Committee for Middle Norway.

### Compliance

Compliance will be assessed based on patients' record of compliance as well as serum control at week 3.

### Neuropsychological assessment

The following tests will be used: Brief Assessment of Cognition in Schizophrenia (BACS), Continuous Performance Test-Identical Pairs (CPT-IP), Wechsler Memory Scale^®^-3rd Ed. (WMS^®^-III): Spatial Span, Letter-Number Span, Hopkins Verbal Learning Test-Revised™ (HVLT-R™), Brief Visuospatial Memory Test-Revised (BVMT-R™), Neuropsychological Assessment Battery^® ^(NAB^®^): Mazes, National Adult Reading Test (NART), The Wechsler Abbreviated Scales of Intelligence (WASI), Autobiographical Memory Interview-Short Form (AMI-SF), The Mini-Mental State (MMS).

### Description of measures

*The MINI *[36] covers 18 Axis I disorders and has shown good inter-rater reliability. The MINI has systematic questions covering the various diagnostic criteria of the Axis I disorders. Since many Axis I disorders demand certain obligatory criteria, the MINI has skipping rules if such criteria are not met. Dimensional criteria scores for various disorders, therefore, cannot be established with the MINI. The rating of each criterion on the MINI is absent or present, and the number of positive criteria is summarized as disorder present or absent. The MINI is a relatively brief structured interview, acceptable for the time-frame of the baseline examination.

*The SCID-I *[37] is a semi structured interview for making the major DSM-IV Axis I diagnoses. The SCID is broken down into separate modules corresponding to categories of diagnoses. Most sections begin with an entry question that would allow the interviewer to "skip" the associated questions if not met. For all diagnoses symptoms are coded as present, subthreshold, or absent.

*Affective symptom ratings: The MADRS *[40] is a short and reliable scale devised to be sensitive to change. It is in daily use by both psychiatrists and general practitioners in Norway. The psychometrics (reliability and validity) of MADRS has been shown to be satisfactory. Patients are rated on ten items, each of which has value ranges from 0 (the least pathology) to 6 (the most sever pathology). Sum scores range from 0 to 60, with a scoring of 20 indicating moderate and 30 severe depression [49]. The scale is sensitive to change and covers many, but not all, symptom domains in depression. The MADRS is among the most frequently used depression rating scales in clinical depression trials.

*The IDS *[41] is a well validated 30-item scale translated into many languages. IDS is devised to cover all symptom domains needed for DSM-IV diagnosis. It also comprises symptoms of melancholia and atypical depression and has scaling items allowing for detection of milder levels of symptoms. It has a more balanced weighting of items than the Hamilton Depression Rating Scale and comprises many more symptom domains than the MADRS. IDS is as sensitive as the Hamilton scale and CGI in detecting between group differences.

*The CGI-BP *[42] is a modification of the CGI specifically for use in assessing global illness severity and change in patients with BD. The revised scale and manual provide a focused set of instructions to facilitate the reliability of these ratings of mania, depression, and overall bipolar illness during treatment of an acute episode or in longer-term illness prophylaxis. The modified CGI-BP is anticipated to be more useful than the original CGI in studies of BD. The clinician forms a global judgement both of the severity of the illness as compared to other cases with the same diagnosis and the global degree of change during treatment. Both sub-scales have value ranges from 1 (best) to 7 (worst).

*The PGI-I *requires the patient to rate how much the illness has improved or worsened relative to a baseline state. The illness is compared to change over time and rated as: very much improved, much improved, minimally improved, no change, minimally worse, much worse, or very much worse.

*The SF-36 *[45] is a multi-purpose, short-form health survey with 36 questions. It yields an eight-scale profile of functional health and well-being scores as well as psychometrically-based physical and mental health summary measures, and a preference-based health utility index. It is a generic measure, as opposed to one that targets a specific age, disease, or treatment group. Accordingly, the SF-36 has proven useful in surveys of general and specific populations, comparing the relative burden of diseases, and in differentiating the health benefits produced by a wide range of different treatments.

*The YMRS *[43] is an 11-item scale used to assess the severity of mania. It takes 15-30 minutes to complete. The YMRS has been used in clinical practice since 1978. Ratings are based on self-reporting and clinician observation.

*The MSIF *[46] is a new instrument for rating functional disability in psychiatric outpatients. The MSIF provides discrete ratings of role responsibility, presence and level of support, and performance quality. The MSIF consists of a semistructured interview and detailed rating anchors. The MSIF is an instrument designed to circumvent several limitations with existing functional outcome instruments for longitudinal studies.

*The TEMPS-A *[47] is a self-report questionnaire designed to measure temperamental variations in psychiatric patients and healthy volunteers. Its constituent subscales and items were formulated on the basis of the diagnostic criteria for affective temperaments (cyclothymic, dysthymic, irritable, hyperthymic, and anxious), originally developed by Akiskal [47].

*Everyday memory questionnaire (EMQ) *[50] is a valid and reliable self-report measure of common memory lapses in everyday activities comprising of 27 statements. Examples included "telling someone a story or joke that you have told them once already" and "forgetting where things are normally kept or looking in the wrong place for them". Responses were on a nine-point scale ranging from 'Not at all in the last six months' to 'More than once a day', with high scores indicating more forgetting.

## Adverse events

### Definitions

#### Adverse event (AE)

Any untoward medical occurrence in a patient that may present itself during treatment or administration with a pharmaceutical product, and which may or may not have a causal relationship with the treatment. An AE can therefore be any unfavourable and unintended sign (including an abnormal finding), symptom or disease temporally associated with the use of a medicinal (investigational) product, whether or not related to the medicinal (investigational) product.

#### Serious Adverse Event (SAE)

Any untoward medical occurrence that at any dose:

- results in death,

- is life-threatening,

- requires inpatient hospitalization or prolongation of existing hospitalization,

- results in persistent or significant disability/incapacity or

- is a congenital anomaly/birth defect

#### Unexpected Adverse Event

Any adverse experience associated with the use of the drug/device, the specificity or severity of which is not consistent with the current Summary of Product Characteristics (SPC); or the specificity or severity of which is not consistent with the risk information provided to subjects (in the Informed Consent Document).

#### Attribution to an investigational agent/procedure

Unrelated: The AE is clearly not related to investigational agents.

Unlikely: The AE is doubtfully related to investigational agents.

Possible: The AE may be related to the investigational agents.

Probable: The AE is likely related to the investigational agents.

Definite: The AE is clearly related to the investigational agents.

### Reporting of adverse events

AEs will be reported starting with the first trial related procedure. They will be reported until intake of the last dose of trial medication or last trial-related procedure. When known, the cause of death of a subject in a clinical trial must be reported as a SAE regardless of whether the event is expected or associated with the study medicines or procedures. All SAE occurring during the clinical trial must be reported to the Clinical Study Team Leader (UK) or principal investigator (GM) by investigational staff within 24 h. Information regarding SAEs must be reported using the SAE form. The report of a SAE may be made by fax or telephone. A telephone report must be followed by a completed SAE Form.

CATEGORY 1: No change required. This category would be for SAEs which in the estimation of the investigators and the RSA that any relationship to the study intervention is questionable and the chances for harm to the future research subjects is unlikely.

CATEGORY 2: Increased monitoring necessary. This category would be for SAEs in which the RSA Committee would require increased frequency or intensity of monitoring.

CATEGORY 3: Intervention or treatment modifications necessary. This category would be for SAEs that would lead to a recommendation to modify the treatment, for example, lowering the dose of the study drug, or extending the treatment interval.

CATEGORY 4: Temporary or permanent suspension of the study. This category would be for SAEs which are judged to be serious enough that no further subjects should be enrolled or treated until a decision about whether the protocol can continue under some modified circumstances. In order to permanently suspend a protocol, a full review of the events by the RSA Committee and the GCRC Advisory Committee will be undertaken.

### Assessment of side effects

The Udvalg for Kliniske Undersøgelser (UKU) Side Effect Rating Scale [51] is used for registration of side effects. UKU is a clinician-rated scale with well-defined and operationalized items to assess the side effects of psychopharmacological medications. The rating is performed by a trained mental health professional on the basis of an interview with the patient and other relevant information from all available sources. In case of discrepancies among the reports, the clinician's observations are given more weight than patient reports. UKU is designed for use in both clinical trials and routine clinical practice. Rating is independent of whether the symptom is regarded as being drug-induced or not. Probability of the causal relationship (or lack of it) of each item to the medication in question is indicated in a separate column, which makes it useful for determining subsequent course of action. Subscales can be useful in assessing differential side effect profiles.

### Emergency procedures

We have procedures for medical emergency:

#### The study emergency contact procedure

In the case of a medical emergency, contact the Clinical Study Team Leader. If the clinical Study Team Leader is not available, contact the principal study investigator, at the Haukeland University hospital or St. Olav's Hospital, Trondheim (Table 5).

**Table 5:** Contact information in case of a medical emergency

Role in the study	Name	Address and Telephone number
Clinical Study Team Leader	Ute Kessler	Haukeland University Hospital, 5021 Bergen, 004755974580
Principle investigator	Gunnar Morken	St. Olav's Hospital, Trondheim, 004773864600

#### Procedures in case of overdose

For the purpose of this study, all overdoses, with or without associated symptoms, should be reported as AEs. However, all cases of overdose must be reported immediately, within 1 day, if sequela meeting the criteria for a SAE have occurred in association with the overdose. In all instances, the overdose substance must be stated and an assessment whether the overdose was accidental or intentional should be recorded. If the overdose was a suicide attempt, this fact should be clearly stated. AE (serious and non-serious) that occur as a result of an overdose should be recorded on the Case report form (CRF) as "sequelae to overdose" (for example, nausea as sequelae to overdose").

#### Procedures in case of suicide attempt and suicide

Suicide and suicide attempt, irrespective of the method, but in connection with the use of study medicine, should be reported as an AE or a SAE in accordance with the definition provided in section 10. This event should be identified as suicide or suicide attempt, and the method of the suicide or the suicide attempt should be provided.

#### Procedures in case of pregnancy

Pregnancy itself is not regarded as an AE unless there is a suspicion that the study medicine may have interfered with the effectiveness of a contraceptive medication. However, the outcome of all pregnancies (spontaneous miscarriage, elective termination, normal birth or congenital abnormality) must be followed up and documented even if the patient was discontinued from the study.

All reports of congenital abnormalities/birth defects are SAEs. Spontaneous miscarriages should also be reported and handles as SAEs. Elective abortions without complications should not be handled as AEs. All outcomes of pregnancy must be reported to the principal investigator.

Female patients of childbearing potential must be using a reliable method of contraception. Reliable methods of contraception include hormonal contraceptives (e.g. oral contraceptives or long term injectable or implantable hormonal contraceptive), double-barrier methods (e.g. condom and diaphragm, condom and foam, condom and sponge), intrauterine devices and tubal ligation. Female patients must have a negative urine human chorionic gonadotropin (HCG) test at enrolment.

## Protocol Deviations

All important deviations related to study inclusion or exclusion criteria, conduct of the

trial, patient managements or patient assessment will be described.

Protocol deviations will be categorized as:

Those who entered the study even though they did not satisfy the entry criteria.

Those who developed withdrawal criteria during the study but were not withdrawn.

Those who received the wrong treatment or incorrect dose.

Those who received excluded concomitant treatment.

## Ethical considerations

### Ethics, Data Security and Biobank

The study will be organized as a subproject in the BRAIN study, which is approved by the Regional Committee for Medical Research Ethics and the Norwegian Data Inspectorate. The bio bank is organized together with the Norwegian TOP (Thematic Organized Psychosis Research) study and has been approved by the Health Inspectorate. The bio bank is located at the National Institute of Health and the Database and protocol for the Person Data Security is approved by the Norwegian Data Inspectorate. There is a separate consent for this intervention study, which is also approved by the Regional Committee for Medical Research Ethics.

The patients included are severely ill, and in need of treatment considered to be effective in treatment resistant depression. ECT and the combination of antidepressant (including irreversible MAOI), atypical antipsychotics and a mood stabilizer are considered to be the treatment of choice in these patients. This means that both patient groups will receive adequate antidepressant treatment for their disorder, but will have a superior clinical management to patients not participating in a study. As stated in the exclusion criteria, some patients (e.g. acutely suicidal patients, post partum depression) should be offered ECT directly.

### Ethical Conduct of the Study

The study is conducted in accordance with the ethical principles that have their origins in the Declaration of Helsinki.

### Patient Information and Consent

Informed consent will be obtained after the investigator has verbally explained the purpose and procedures involved in the study, answered questions, and otherwise provided information that permits the patient to make a prospective, informed decision. Informed consent will be signed and dated before any study data collection procedures begin.

A synopsis of the study protocol is given as additional file 2.

## Discussion

This study protocol presents a randomized controlled trial for a population of in-patients with serious, therapy resistant bipolar depressions. We believe that this study is important and needed, and that the main results may influence daily clinical practise. Both bipolar depression and the treatments for bipolar depression are associated with cognitive impairments. This study may give important information of short-and long-time contributions and effects of these different factors.

### Methodological strengths

The present study is strengthened by the prospective design. All participating study centres include patients from their defined catchment areas. All Norwegian acute psychiatric services are public and available to everyone. All the patients in the catchment areas are admitted to the local study centre. Acute admissions to other psychiatric hospitals occur only when inhabitants temporarily reside outside of the catchment area at the time of admittance.

We use robust and validated diagnostic and psychometric instruments. Both in the study and control groups the procedures and registrations for each patient are comprehensive. The collection of background data with the NEQ makes it possible to compare the present bipolar population with bipolar populations in other clinical trials.

### Methodological weaknesses

The use and policy regarding ECT differs between countries and hospitals. Thus there are no published guidelines defining objective clinical criteria for indications of treatment with ECT in bipolar depressions. In the present protocol "indication for ECT" is one of the inclusion criteria. This means that treatment with ECT would have been probable according to hospital practice regardless of the present study.

The assessments are performed by specially trained research staff which is not blinded for grouping of the patients. Most of the patients are acutely admitted to psychiatric acute wards due to the extensive symptom pressure.

The multicentre site design has potential problems regarding assessments and inter-rater reliability. However, multicentre design is a necessity in order to include sufficient number of patients in a reasonable time span. All raters and participants in the study have taken part in mutual training-sessions in the different assessments and rating scales. All participants have taken part in interrater-scorings. The scorings for the different study centres and the different participants are satisfactory.

## Competing interests

UK has received travel support from Lundbeck in 2007. AV has received research support from GlaxoSmithKline. HS has received speaker honorarium from Eli Lilly and Pfizer, and travel support from Eli Lilly, Lundbeck, Janssen-Cilag and Astra Zeneca. KJO has received speaker honorarium or travel support from Lundbeck, Astra Zeneca, Bristol-Myer Squibbs, Pfizer, Wyeth, Eli Lilly, Janssen-Cilag and Desitin and research support from Eli Lilly and Lundbeck and been the Principal Investigator of a Clinical Trial sponsored by Astra Zeneca. PB has received travel supports from different pharmaceutical companies to attend conferences with various psychiatric topics. He has been invited as a lecturer by AstraZeneca, Eli Lilly, GlaxoSmithKline, Lundbeck, Organon, Pfizer, Sanofi-Synthelabo and Wyeth. OAA has received speaker honorarium and travel support from Lundbeck, Astra Zeneca, Bristol-Myer Squibbs, Pfizer, Wyeth, Eli Lilly, and Janssen-Cilag, and research support from Eli Lilly. UFM has been given fees for lecturing for Astra Zeneca, Bristol Myers Squibb, Glaxo Smith Kline, Lilly, Lundbeck, MSD (Organon), and Wyeth. His research group has been given an unrestricted research grant from Lundbeck. His spouse worked as a medical advisor for Pfizer Norway until 2010. GM received travel support from Astra Zeneca in 2007.

## Authors' contributions

UK, HS, KJO, PB, OAA, UFM and GM contributed to the background and design of the study. UK and AV drafted the manuscript. All the authors read and approved the final manuscript.

## Pre-publication history

The pre-publication history for this paper can be accessed here:

http://www.biomedcentral.com/1471-244X/10/16/prepub

## Supplementary Material

Additional file 1 **Treatment algorithm for control group**. Treatment algorithm for control group Click here for file

Additional file 2 **Protocol synopsis. **Gives an overview of the study protocol Click here for file

## References

[B1] Kessler RC McGonagle KA Zhao S Nelson CB Hughes M Eshleman S Wittchen HU Kendler KS Lifetime and 12-month prevalence of DSM-III-R psychiatric disorders in the United States. Results from the National Comorbidity Survey Arch Gen Psychiatry 1994 51 1 8 19 8279933 10.1001/archpsyc.1994.03950010008002

[B2] Simon GE Ludman EJ Bauer MS Unutzer J Operskalski B Long-term effectiveness and cost of a systematic care program for bipolar disorder Arch Gen Psychiatry 2006 63 5 500 508 10.1001/archpsyc.63.5.500 16651507

[B3] Judd LL Schettler PJ Akiskal HS Maser J Coryell W Solomon D Endicott J Keller M Long-term symptomatic status of bipolar I vs. bipolar II disorders Int J Neuropsychopharmacol 2003 6 2 127 137 10.1017/S1461145703003341 12890306

[B4] Post RM Denicoff KD Leverich GS Altshuler LL Frye MA Suppes TM Rush AJ Keck PE Jr McElroy SL Luckenbaugh DA Pollio C Kupka R Nolen WA Morbidity in 258 bipolar outpatients followed for 1 year with daily prospective ratings on the NIMH life chart method J Clin Psychiatry 2003 64 6 680 690 quiz 738-689 12823083 10.4088/jcp.v64n0610

[B5] Thase ME Bipolar depression: issues in diagnosis and treatment Harv Rev Psychiatry 2005 13 5 257 271 10.1080/10673220500326425 16251165

[B6] Sachs GS Nierenberg AA Calabrese JR Marangell LB Wisniewski SR Gyulai L Friedman ES Bowden CL Fossey MD Ostacher MJ Ketter TA Patel J Hauser P Rapport D Martinez JM Allen MH Miklowitz DJ Otto MW Dennehy EB Thase ME Effectiveness of adjunctive antidepressant treatment for bipolar depression N Engl J Med 2007 356 17 1711 1722 10.1056/NEJMoa064135 17392295

[B7] Geddes JR Burgess S Hawton K Jamison K Goodwin GM Long-term lithium therapy for bipolar disorder: systematic review and meta-analysis of randomized controlled trials Am J Psychiatry 2004 161 2 217 222 10.1176/appi.ajp.161.2.217 14754766

[B8] Tohen M Vieta E Calabrese J Ketter TA Sachs G Bowden C Mitchell PB Centorrino F Risser R Baker RW Evans AR Beymer K Dube S Tollefson GD Breier A Efficacy of olanzapine and olanzapine-fluoxetine combination in the treatment of bipolar I depression Arch Gen Psychiatry 2003 60 11 1079 1088 10.1001/archpsyc.60.11.1079 14609883

[B9] Calabrese JR Keck PE Jr Macfadden W Minkwitz M Ketter TA Weisler RH Cutler AJ McCoy R Wilson E Mullen J A randomized, double-blind, placebo-controlled trial of quetiapine in the treatment of bipolar I or II depression Am J Psychiatry 2005 162 7 1351 1360 10.1176/appi.ajp.162.7.1351 15994719

[B10] Geddes JR Calabrese JR Goodwin GM Lamotrigine for treatment of bipolar depression: independent meta-analysis and meta-regression of individual patient data from five randomised trials Br J Psychiatry 2009 194 1 4 9 10.1192/bjp.bp.107.048504 19118318

[B11] Hirschfeld RMA Bowden CL Gitlin MJ Keck PE Suppes T Thase ME Wagner KD Perlis RH American Psychiatric Association: Practice Guidelines for the treatment of patients with bipolar disorder 2002 2 Washington DC: American Psychiatric Association

[B12] Moksnes KM Vatnaland T Eri B Torvik NH Electroconvulsive therapy in the Ullevaal region of Oslo 1988-2002 Tidsskr Nor Laegeforen 2006 126 13 1750 1753 16794670

[B13] Weiner RD Coffey CE Fochtmann LJ Greenberg RM Isenberg KE Kellner CH Sackeim HA Moench L The Practice of Electroconvulsive Therapy. Recommendations for Treatment, Training, and Privileging: A Task Force Report of the American Psychiatric Association 2001 2 Washington: American Psychiatric Association

[B14] Sienaert P Peuskens J Electroconvulsive therapy: an effective therapy of medication-resistant bipolar disorder Bipolar Disord 2006 8 3 304 306 10.1111/j.1399-5618.2006.00317.x 16696836

[B15] Souery D Papakostas GI Trivedi MH Treatment-resistant depression J Clin Psychiatry 2006 67 Suppl 6 16 22 16848672

[B16] Sweeney JA Kmiec JA Kupfer DJ Neuropsychologic impairments in bipolar and unipolar mood disorders on the CANTAB neurocognitive battery Biol Psychiatry 2000 48 7 674 684 10.1016/S0006-3223(00)00910-0 11032979

[B17] Holmes MK Erickson K Luckenbaugh DA Drevets WC Bain EE Cannon DM Snow J Sahakian BJ Manji HK Zarate CA Jr A comparison of cognitive functioning in medicated and unmedicated subjects with bipolar depression Bipolar Disord 2008 10 7 806 815 10.1111/j.1399-5618.2008.00628.x 19032712 PMC2727596

[B18] Quraishi S Frangou S Neuropsychology of bipolar disorder: a review J Affect Disord 2002 72 3 209 226 10.1016/S0165-0327(02)00091-5 12450638

[B19] McCall WV Reboussin DM Weiner RD Sackeim HA Titrated moderately suprathreshold vs fixed high-dose right unilateral electroconvulsive therapy: acute antidepressant and cognitive effects Arch Gen Psychiatry 2000 57 5 438 444 10.1001/archpsyc.57.5.438 10807483

[B20] Sackeim HA Prudic J Devanand DP Nobler MS Lisanby SH Peyser S Fitzsimons L Moody BJ Clark J A prospective, randomized, double-blind comparison of bilateral and right unilateral electroconvulsive therapy at different stimulus intensities Arch Gen Psychiatry 2000 57 5 425 434 10.1001/archpsyc.57.5.425 10807482

[B21] Sackeim HA Prudic J Fuller R Keilp J Lavori PW Olfson M The cognitive effects of electroconvulsive therapy in community settings Neuropsychopharmacology 2007 32 1 244 254 10.1038/sj.npp.1301180 16936712

[B22] Stern Y Sano M Pauson J Mayeux R Modified Mini-Mental State Examination: validity and reliability Neurology 1987 37 1793808297

[B23] National Institute for Clinical Excellence. Guidance on the Use of Electroconvulsive Therapy 2003 London

[B24] Anisman H Merali Z Cytokines, stress and depressive illness: brain-immune interactions Ann Med 2003 35 1 2 11 10.1080/07853890310004075 12693607

[B25] Capuron L Dantzer R Cytokines and depression: the need for a new paradigm Brain Behav Immun 2003 17 Suppl 1 S119 124 10.1016/S0889-1591(02)00078-8 12615197

[B26] Sinha G Inflamed brains can trigger the blues, studies suggest Nat Med 2004 10 10 1007 10.1038/nm1004-1007b 15459686

[B27] Dunn AJ Swiergiel AH de Beaurepaire R Cytokines as mediators of depression: what can we learn from animal studies? Neurosci Biobehav Rev 2005 29 4-5 891 909 10.1016/j.neubiorev.2005.03.023 15885777

[B28] O'Brien SM Scully P Scott LV Dinan TG Cytokine profiles in bipolar affective disorder: focus on acutely ill patients J Affect Disord 2006 90 2-3 263 267 10.1016/j.jad.2005.11.015 16410025

[B29] Goldstein BI Kemp DE Soczynska JK McIntyre RS Inflammation and the phenomenology, pathophysiology, comorbidity, and treatment of bipolar disorder: a systematic review of the literature J Clin Psychiatry 2009 70 8 1078 1090 10.4088/JCP.08r04505 19497250

[B30] Soczynska JK Kennedy SH Goldstein BI Lachowski A Woldeyohannes HO McIntyre RS The effect of tumor necrosis factor antagonists on mood and mental health-associated quality of life: Novel hypothesis-driven treatments for bipolar depression? Neurotoxicology 2009 30 4 497 521 10.1016/j.neuro.2009.03.004 19477018

[B31] Goodwin FK Jamison KR Goodwin FK, Jamison KR Maintenance Medical Treatment Manic-Depressive Illness: Bipolar Disorders and Recurrent Depression 2007 2 New York: Oxford University Press 797 848

[B32] d'Elia G Unilateral electroconvulsive therapy Acta Psychiatr Scand Suppl 1970 215 1 98 5271208

[B33] Abrams R Electroconvulsive therapy 2002 4 New York: Oxford University Press

[B34] d'Elia G Present Practice of Electroconvulsive Therapy in Scandinavia Arch Gen Psychiatry 1983 40 577 581 6838335 10.1001/archpsyc.1983.01790050103013

[B35] Goodwin FK Jamison KR Goodwin FK, Jamison KR Medical Treatment of Depression Manic-Depressive Illness: Bipolar Disorders and Recurrent Depression 2007 2 New York: Oxford University Press 752 753

[B36] Sheehan DV Lecrubier Y Sheehan KH Amorim P Janavs J Weiller E Hergueta T Baker R Dunbar GC The Mini-International Neuropsychiatric Interview (M.I.N.I.): the development and validation of a structured diagnostic psychiatric interview for DSM-IV and ICD-10 J Clin Psychiatry 1998 59 Suppl 20 22 33 quiz 34-57 9881538

[B37] First M Spitzer R Gibbon M Williams J Structured Clinical Interview for DSM-IV-TR Axis I Disorders, Research Version, Non-patient Edition. (SCID-I/NP) 2002 New York: Biometrics Research, New York State Psychiatric Institute

[B38] Post RM Nolen WA Kupka RW Denicoff KD Leverich GS Keck PE Jr McElroy SL Rush AJ Suppes T Altshuler LL Frye MA Grunze H Walden J The Stanley Foundation Bipolar Network I. Rationale and Methods Br J Psychiatry 2001 178 suppl 41 169 176 10.1192/bjp.178.41.s169 11450179

[B39] Leverich GS Nolen WA Rush AJ McElroy SL Keck PE Denicoff KD Suppes T Altshuler LL Kupka R Kramlinger KG Post RM The Stanley Foundation Bipolar Treatment Outcome Network. I. Longitudinal methodology J Affect Disord 2001 67 1-3 33 44 10.1016/S0165-0327(01)00430-X 11869751

[B40] Montgomery SA Asberg M A new depression scale designed to be sensitive to change Br J Psychiatry 1979 134 382 389 10.1192/bjp.134.4.382 444788

[B41] Rush AJ Giles DE Schlesser MA Fulton CL Weissenburger J Burns C The Inventory for Depressive Symptomatology (IDS): preliminary findings Psychiatry Res 1986 18 1 65 87 10.1016/0165-1781(86)90060-0 3737788

[B42] Spearing MK Post RM Leverich GS Brandt D Nolen W Modification of the Clinical Global Impressions (CGI) Scale for use in bipolar illness (BP): the CGI-BP Psychiatry Res 1997 73 3 159 171 10.1016/S0165-1781(97)00123-6 9481807

[B43] Young RC Biggs JT Ziegler VE Meyer DA A rating scale for mania: reliability, validity and sensitivity Br J Psychiatry 1978 133 429 435 10.1192/bjp.133.5.429 728692

[B44] Jones SH Thornicroft G Coffey M Dunn G A brief mental health outcome scale-reliability and validity of the Global Assessment of Functioning (GAF) Br J Psychiatry 1995 166 5 654 659 10.1192/bjp.166.5.654 7620753

[B45] Ware JE Jr Sherbourne CD The MOS 36-item short-form health survey (SF-36). I. Conceptual framework and item selection Med Care 1992 30 6 473 483 10.1097/00005650-199206000-00002 1593914

[B46] Berns S Uzelac S Gonzalez C Jaeger J Methodological considerations of measuring disability in bipolar disorder: validity of the Multidimensional Scale of Independent Functioning Bipolar Disord 2007 9 1-2 3 10 10.1111/j.1399-5618.2007.00305.x 17391344

[B47] Akiskal HS Akiskal KK Haykal RF Manning JS Connor PD TEMPS-A: progress towards validation of a self-rated clinical version of the Temperament Evaluation of the Memphis, Pisa, Paris, and San Diego Autoquestionnaire J Affect Disord 2005 85 1-2 3 16 10.1016/j.jad.2004.12.001 15780671

[B48] Low NC Du Fort GG Cervantes P Prevalence, clinical correlates, and treatment of migraine in bipolar disorder Headache 2003 43 9 940 949 10.1046/j.1526-4610.2003.03184.x 14511270

[B49] Mulder RT Joyce PR Frampton C Relationships among measures of treatment outcome in depressed patients J Affect Disord 2003 76 1-3 127 135 10.1016/S0165-0327(02)00080-0 12943942

[B50] Sunderland A Harri JE Baddeley AD Do laboratory tests predict everyday memory? J Verb Learn Verb Behav 1983 22 341 357 10.1016/S0022-5371(83)90229-3

[B51] Lingjaerde O Ahlfors U Bech P Dencker S Elgen K The UKU side effect rating scale. A new comprehensive rating scale for psychotropic drugs and a cross-sectional study of side effects in neuroleptic-treated patients Acta Psychiatr Scand Suppl 1987 334 1 100 10.1111/j.1600-0447.1987.tb10566.x 2887090

[B52] Diagnostic and Statistical Manual of Mental Disorders, Fourth Edition, Text Revision (DSM-IV-TR) 2000 10.1111/j.1743-6109.2007.00557.x 17727354

[B53] Angst J Adolfsson R Benazzi F Gamma A Hantouche E Meyer TD Skeppar P Vieta E Scott J The HCL-32: towards a self-assessment tool for hypomanic symptoms in outpatients J Affect Disord 2005 88 2 217 233 10.1016/j.jad.2005.05.011 16125784

