# Supplementary material for: The study protocol of the Norwegian randomized controlled trial of electroconvulsive therapy in treatment resistant depression in bipolar disorder
Source: BMC Psychiatry. 2010 Feb 23;10:16. doi: 10.1186/1471-244X-10-16 (PMC2847998; doi:10.1186/1471-244X-10-16)
Supplement: Additional file 1 — Treatment algorithm for control group. Treatment algorithm for control group [file 1471-244X-10-16-S1.DOC]

**Additional file 1 - Treatment algorithm for control group**

There exist several treatment algorithms for bipolar depression. The algorithm by Goodwin and Jamison [35] from 2007 is one of the most updated.

This is further simplified to match the Norwegian clinical practice.

In accordance with the evidence, the algorithm is separated in treatment of bipolar I or bipolar II depression, and the treatment advice depends if the patients are on a mood stabilizer or not.

The treatment suggestions are divided in different steps, starting with step 1. If the patient has used the medications suggested in step 1 in lifetime without effect on a depressive episode, proceed to the next step in the algorithm. The chosen medical intervention is to be continued trough the six weeks study phase.

Goodwin and Jamison 2007 [35] underscores the importance of evaluating the thyroid function in all patients and supplement with thyroid hormones if FT4 or TSH is outside the 50-percentile, even though within the “normal range”. Even within the 50-percentile T3 or T4 may increase response of psychopharmacological therapies, without side effects.

***Treatment of Bipolar-I Depression***

| If not on a mood stabilizer | If on lithium or valproate |
| --- | --- |
| Step 1:  Start lamotrigine combined with lithium or valproate.  For severe depression, consider an antidepressant plus an antimanic mood stabilizer*.  For psychotic depression, add an atypical. | Step 1:  If on lithium, increase dose**.  Add lamotrigine. |
| Step 2:  Add quetiapine. | Step 2:  Add quetiapine. |
| Step 3:  Consider olanzapine plus fluoxetine as an alternative to quetiapine. | Step 3:  Consider olanzapine plus fluoxetine as an alternative to quetiapine. |
| Step 4:  Discontinue olanzapine plus fluoxetine and add an antidepressant, while maximizing the antimanic mood stabilizer. | Step 4:  Discontinue olanzapine plus fluoxetine and add an antidepressant, while maximizing the antimanic mood stabilizer. |

*Antimanic mood stabilizers: lithium, valproate, carbamazepin, oxcarbazepin and atypical antipsychotics.

** Increase lithium to preferably above 0.8, but not above 1.2 mEq/l in serum concentration.

Antidepressants can be referred to as first-generations and 2. Generations antidepressants (ATC nr N06 A) and Parnate (Tranylcypromide sulfate) and Nardil (Phenelzine), which have no ATC nr.

Antipsychotics according to the algorithm are Olanzapin and Quetiapine (ATC nr N05 AH, 03 and 04 respectively).

Mood stabilizers are of different kinds; e.g. anticonvulsants: (Carbamazepin, Oxcarbazepin, Vaproate and Lamotrigine (N03 AF, N03 AG01 and N03 AX09)) and Lithium (ATC nr N05AN01) and Litarex.

Treatment of Bipolar-II Depression

| If not on a mood stabilizer | If on a mood stabilizer |
| --- | --- |
| Step 1:  Start lamotrigine.  For severe depression consider an antidepressant plus an antimanic mood stabilizer*.  For psychotic depression, add an atypical antipsychotic to lamotrigine.  For persistent irritability, consider adjunctive valproate. | Step 1:  If on lithium, increase dose**.  Add lamotrigine. |
| Step 2:  Consider a second generation antidepressant plus an antimanic mood stabilizer or quetiapine. | Step 2:  Consider a second-generation antidepressant plus an antimanic mood stabilizer or quetiapine. |
| Step 3:  Consider combinations of two mood stabilizers or of one mood stabilizer and an antidepressant. | Step 3:  Consider combinations of two mood stabilizers or of one mood stabilizer and an antidepressant. |

*Antimanic mood stabilizers: lithium, valproate, carbamazepin, oxcarbazepin and atypical antipsychotics.

** Increase lithium to preferably above 0.8, but not above 1.2 mEq/lin serum concentration.

Antidepressants can be referred to as first- generation and second- generation antidepressants (ATC nr N06 A) and Parnate (Tranylcypromide sulfate) and Nardil (Phenelzine), which have no ATC nr.

Antipsychotics according to the algorithm are Olanzapin and Quetiapine (ATC nr N05 AH, 03 and 04 respectively).

Mood stabilizers are of different kinds; e.g. anticonvulsants: (Carbamazepin, oxcarbazepin, Vaproate and Lamotrigine (N03 AF, N03 AG01 and N03 AX09)) and lithium (ATC nr N05AN01) and Litarex.
