# Supplementary material for: The study protocol of the Norwegian randomized controlled trial of electroconvulsive therapy in treatment resistant depression in bipolar disorder
Source: BMC Psychiatry. 2010 Feb 23;10:16. doi: 10.1186/1471-244X-10-16 (PMC2847998; doi:10.1186/1471-244X-10-16)
Supplement: Additional file 2 — Protocol synopsis. Gives an overview of the study protocol [file 1471-244X-10-16-S2.DOC]

**Additional file 2** - Protocol synopsis

| EudraCT number:  2007-007175-16 |
| --- |
| Funding: An investigator- initiated trial financed by Helse Vest and Norwegian Hospitals |
| Principle Investigator: Gunnar Morken, St. Olavs University Hospital, NTNU, Trondheim, Norway |
| Objectives: Compare the effect of Electro Convulsive Therapy (ECT) with drug treatment of depression in BD and investigate cognitive changes during the treatment. |
| Study design: Prospective, randomised controlled multi-centre trial |
| Method: 6- week acute treatment trial with 7 clinical assessments. Follow up visit after 26 weeks and between 26 and 52 weeks for those patients who where not in euthymic state at week 26. |
| Number of Subjects Planned: 132  The main outcome parameter is change in the MADRS score. The study will have a 90 % chance of detecting a difference of 4 points in change on the MADRS with a significance level of 0.05. A 4 point difference in change on the MADRS is a moderate effect size and is considered clinically significant. |
| Diagnosis and Main Criteria for Inclusion: Treatment resistant depression in BD, MADRS score of at least 25 at baseline |
| Investigational Product, Dose and Mode of Administration:  ECT group: 3 sessions per week for up to 6 weeks, total up to 18 sessions.  Comparison group: algorithm-based treatment as usual |
| Study Duration: 2008-2020 |
| Criteria for Evaluation:  Efficacy endpoints:  Change in level of depression, measured with MADRS  Cognitive functioning, measured with Neuropsychological tests.  Safety endpoints:  Worsening of symptoms, suicidality, side effect |
| Statistical Methods:  Response and remission rates will be compared using two-sample t-test. Secondary outcomes and cognitive function will be analysed with multiple regression analyses and other suitable statistical methods.  We will use last observations carried forward for subjects who leave the study prematurely. |
| Centers/(Clinical Investigators, co-investigators): Haukeland University Hospital, Bergen/(Ute Kessler, Ketil Ødegård, Jorunn Torgauten); Stavanger University Hospital /(Helle Schøyen, Trine Grønning); Psychiatric Clinic Førde /(Per Bergsholm); Division of Psychiatry, Ullevål University Hospital Oslo, Acute Unit/ (Paul Stronegger, Ole Andreassen); Division of Psychiatry, Ullevål University Hospital Oslo, Gerontopsychiatric Unit/ (Kjell M. Moksness ); Clinic of Psychiatry, Aker University Hospital/ (Harald Brauer); Clinic of Psychiatry, Østfold County Hospital Fredrikstad/ (Jarle B. Johansen, Christian Fahraeus); Clinic of Psychiatry, Levanger/ (Nils H. Dahl); Østmarka Psychiatric Department, St. Olav University Hospital Trondheim/ (Gunnar Morken, Arne Vaaler) |
| Collaborators: Ass Prof. Dr. Psychol. Åsa Hammar, University of Bergen; Prof. Kjetil Sundet, University of Oslo |
| Steering committee: Prof. Gunnar Morken, Prof. Ulrik Malt, Prof. Ole Andreassen, Ass Prof. Per Bergsholm, Ass Prof. Ketil Ødegård, MD Ute Kessler, MD Helle Schøyen, Dr.med. Arne Vaaler |
